# Steady-state P800 oxidation kinetics indicate that heliobacterial phototrophy is light-limited

**DOI:** 10.1007/s11120-025-01167-0

**Published:** 2025-09-15

**Authors:** Tayleigh Price, Hallie A. C. Chavez, Alysa J. L. Giudici, Alexus Acton, Meaghan Stafford, Steven P. Romberger

**Affiliations:** 1 Biology Department, Hiram College, Hiram, OH 44234 USA; 2 Chemistry Department, Hiram College, Hiram, OH 44234 USA; 3 Present Address: Physician Assistant Program, Baldwin Wallace University, Berea, OH 44017 USA; 4 Present Address: Department of Materials Science & Engineering, Case Western Reserve University, Cleveland, OH 44106 USA; 5 Present Address: ASM International, Novelty, OH 44073 USA; 6 Present Address: College of Veterinary Medicine, Cornell University, Ithaca, NY 14853 USA; 7 Present Address: Medpace Inc, Cincinnati, OH 45277 USA; 8 Present Address: Richard A. Gillespie College of Veterinary Medicine, Lincoln Memorial University, Harrogate, TN 37752 USA

**Keywords:** Heliobacteria, Photosynthesis, Type I reaction center, P800, Steady-state kinetics

## Abstract

The heliobacteria are a family of phototrophic bacteria known for their unique production of bacteriochlorophyll *g* and for their use of the simplest known Type I reaction center. In this work, we characterize P800 oxidation kinetics in whole cells of *Heliomicrobium modesticaldum* under the continuous illumination that is more consistent with in vivo conditions, an area of research that remains largely unexplored. When assayed at 800 nm, whole cells display a large bleaching immediately upon illumination by actinic light, corresponding to the production of P800^+^. The initial bleaching typically reaches a maximum intensity at 10–30 ms, at which point a slower, partial recovery leads to a steady-state that is smaller than the initial bleaching. The effects of charged redox reagents, in particular ferric ammonium citrate, and the cytochrome *bc* complex inhibitor azoxystrobin, demonstrate that this recovery phase is due to forward donation to P800^+^ from cytochrome *c*. A steady-state kinetics analysis comparing the effects of actinic intensity on the rate of P800 oxidation to that of P700 oxidation in spinach chloroplasts and whole cells of *Synechococcus* sp. PCC 7002, suggest that the heliobacterial reaction center is inherently light-limited. In support of a light-limited model, light saturation profiles of untreated cells compared to those treated with ferric ammonium citrate indicated that only 32% of the P800 pool is oxidized during continuous illumination. Taken together, these results indicate that, in stark contrast to all other known phototrophs, phototrophy in the heliobacteria is light-limited.

## Introduction

The heliobacteria are the only known group of phototrophic Gram-positive bacteria, forming a distinct family of four genera in the class *Clostridia* phylum *Firmicutes* (Kyndt et al. 2021). Commonly found in soils, they are strict anaerobes and noted diazotrophs (Asao and Madigan 2010; Kimble and Madigan 1992) but are not obligate phototrophs, capable of both photoheterotrophic growth in the light and chemotrophic growth in the dark (Madigan 2006; Sattley and Blankenship 2010). During phototrophic growth they require either pyruvate, lactate, or acetate (+ CO_2_) as a carbon source, although the use of butyrate (+ CO_2_), ethanol (+ CO_2_) (Madigan 2006), and some six carbon carbohydrates have been reported (Ormerod et al. 1996; Stevenson et al. 1997; Tang et al. 2010). Only pyruvate has been shown to support chemotrophic growth (Kimble et al. 1994, 1995).

The heliobacteria are known for several unique features compared to the other known phototrophs, such as their ability to form endospores (Asao and Madigan 2010; Kimble-Long and Madigan 2001) and for their lack of intracytoplasmic membranes or other higher-level organization of their photosynthetic units. They are also known for their unique production of bacteriochlorophyll (BChl) *g*, an isomer of chlorophyll (Chl) *a* in which the Q_y_ band is red-shifted 100 nm. This structural and spectroscopic shift of their primary pigment imparts a distinct brown color to the organisms and allows harvesting of light in a region of the spectrum that is relatively neglected by other phototrophs (Madigan 2006). Interestingly, BChl *g* is oxygen sensitive, converting to Chl *a* and Chl *a* derivatives upon exposure to both light and oxygen (Brockmann and Lipinski 1983; Beer-Romero et al. 1988; Kobayashi et al. 1998). While complete conversion of the cell’s BChl *g* pool results in a loss of photosynthetic activity, some activity remains even upon conversion of a significant portion of BChl *g* (Ferlez et al. 2015; Thamarath et al. 2012). Thus, the potential drawbacks of oxygen sensitivity are avoided due to the strictly anaerobic nature of the heliobacteria and the resiliency of the system with partial conversion.

Like all chlorophototrophs, the primary photochemical reactions in the heliobacteria occur in a (bacterio)chlorophyll-binding pigment-protein complex termed a reaction center (RC). RCs use the energy of an absorbed photon and a series of electron transfer cofactors to generate and stabilize a charge-separated state. In brief, absorption of a photon results in the net transfer of an electron from a special pair of (B)Chl molecules, termed the primary donor, through a series of bound cofactors to a terminal acceptor. In turn, the reduced terminal acceptor reduces an acceptor molecule that is exogenous to the RC and, likewise, the oxidized primary donor oxidizes an exogenous donor molecule (Bryant and Frigaard 2006; Thiel et al. 2018). Thus, the RC is an enzyme: a light-driven oxidoreductase in which the free-energy of an absorbed photon drives a thermodynamically uphill redox reaction between two substrates, thereby providing the free-energy for downstream metabolic processes involving the oxidized and reduced products. All reaction centers can be divided into two groups depending upon the chemical identity of the terminal electron transfer cofactor. Type II RCs, such as Photosystem II, utilize a quinone as the terminal acceptor whereas Type I RCs, such as Photosystem I, utilize bound [4Fe-4S]^2+/1+^ clusters (Golbeck 1993). Unlike plants and cyanobacteria, in which a Type II RC works in tandem with a Type I RC to reduce a ferredoxin and oxidize water, the heliobacteria utilize a single Type I RC to generate a reduced ferredoxin (Romberger and Golbeck 2012; Walters et al. 2024) and an oxidized cytochrome *c* (Fuller et al. 1985; Redding et al. 2014).

The heliobacterial reaction center (HbRC) is considered the simplest known RC and, in line with many of its phototrophic processes, represents a minimalist approach to a RC. Like the RCs from the chloracidobacteria and green sulfur bacteria, the HbRC is classified as a homodimeric Type I RC (Niederman 2024), consisting of a homodimeric PshA_2_ core and two copies of the small, single alpha helix subunit termed PshX. Thus, the HbRC possesses a total complex stoichiometry of PshA_2_PshX_2_ arranged with a perfect *C*_*2*_ symmetry (Gisriel et al. 2017). The PshA_2_ core forms the bulk of the proteinaceous RC mass and binds the vast majority of cofactors, including the entirety of the electron transfer cofactors and most of the antennae pigments. It is also responsible for binding the membrane-associated cytochrome *c* (cyt *c*) and the cytoplasmically soluble ferredoxin that are the enzymatic substrates of the RC. Each PshX subunit consists of a single, transmembrane alpha helix that binds two BChl *g* molecules (Gisriel et al. 2017). These subunits have recently been shown to help stabilize the structure of the HbRC and to function as low energy antennae, absorbing photons at wavelengths longer than the 800 nm of the P800 primary donor (Orf et al. 2022). The remaining antennae consists of an additional 48 BChl *g*, two BChl *g’* (the C-13^1^ epimer of BChl *g*), and two 4,4’-diaponeurosporene, all of which are bound by the PshA_2_ core (Gisriel et al. 2017). Notably, there are none of the additional subunits found in the homodimeric chloracidobacterial or green sulfur bacterial RCs, such as RC-bound cytochromes, F_A_/F_B_-containing subunits, or antennae complexes.

The electron transfer chain is arranged along the *C*_*2*_ symmetry axis and begins with the P800 primary donor consisting of a special pair of BChl *g’* molecules located near the proposed cyt *c* binding site on the periplasmic side of the membrane. The electron transfer chain then splits into two symmetric branches, each consisting of an accessory BChl *g* followed by an 8^1^-OH Chl *a*_*F*_ molecule (the F subscript denoting a farnesyl tail) that acts as the primary acceptor (termed A_0_). The two branches rejoin at the interpolypeptide [4Fe-4S]^2+/1+^ cluster termed F_X_ which is located near the predicted ferredoxin binding site on the cytoplasmic side of the membrane (Gisriel et al. 2017). Unlike all other known Type I RCs (Jordan et al. 2001; Chen et al. 2020; Dong et al. 2022), the two additional [4Fe-4S]^2+/1+^ clusters termed F_A_ and F_B_ are not present (Gisriel et al. 2017) and F_X_ acts as the terminal electron acceptor (Romberger and Golbeck 2012).

While the initial charge separation events and the specific roles of P800, the accessory BChl *g*, and A_0_ are still under significant investigation (Brütting et al. 2023; Chauvet et al. 2013; Kojima et al. 2020; Orf and Redding 2021; Song et al. 2021), absorption of a photon results in charge separation and electron transfer from P800 to A_0_. The electron is subsequently transferred to F_X_, resulting in formation of the stable P800^+^F_X_^−^ state, which typically has a lifetime of 15–20 ms (Heinnickel et al. 2006). At this point, P800^+^ oxidizes a RC-exogenous membrane-associated cyt *c* (Fuller et al. 1985; Redding et al. 2014) and F_X_^−^ reduces one of several possible ferredoxins (Walters et al. 2024), thereby generating the oxidized cyt *c* and reduced ferredoxin that are the enzymatic products of the RC. The oxidized cyt *c* is subsequently re-reduced by the cytochrome *bc* complex as part of the quinone cycle (Kramer et al. 1997; Leung et al. 2021). The reduced ferredoxin provides the reducing equivalents necessary for other redox enzymes, such as ferrodoxin:NADP oxidoreductase or pyruvate:ferredoxin oxidoreductase (Walters et al. 2024). Like the other homodimeric Type I RCs (Chen et al. 2020; Dong et al. 2022), no quinone molecules are found in the electron transfer chain (Gisriel et al. 2017), which is consistent with the lack of quinone-attributable spectroscopic signals (Brettel et al. 1998; Chauvet et al. 2013) and the finding that HbRCs lacking quinone are fully capable of electron transfer (Kleinherenbrink et al. 1993). However, recent work has pointed to a possible menaquinone binding site located near the primary acceptor and demonstrated light-driven quinone reduction in membranes lacking ferredoxin or other known F_X_ acceptors, suggesting that menaquinone could be acting as part of a secondary electron transfer pathway that is active under high-light conditions (Kashey et al. 2018).

A significant body of work using single-turnover and fluorescence approaches to study isolated membranes and HbRCs has led to a much better understanding of the primary photochemical reactions in the heliobacteria. However, a much smaller body of work has examined these same processes in whole cells under constant illumination, a set of conditions more consistent with in vivo conditions. In this study, we characterize steady-state P800 oxidation kinetics in whole cells of *Heliomicrobium modesticaldum* under constant illumination. Unexpectedly, P800 oxidation shows multiphasic kinetics with a light-induced recovery phase reminiscent of the inductive effect observed with P700 oxidation kinetics in algae and cyanobacteria. Using charged redox reagents and cytochrome *bc* complex inhibitors, we identify the origin of this recovery phase and demonstrate that only a small percentage of the P800 pool is oxidized during constant illumination. A steady-state kinetics analysis of P800 oxidation unexpectedly indicates that an exceedingly high actinic intensity would be required to saturate the rate of P800 oxidation, a finding opposite of that in cyanobacteria and chloroplasts. Taken together, these results suggest that phototrophy in the heliobacteria is light-limited and that they are the only known phototrophs to function in this manner.

## Materials and methods

### Reagents and biochemical manipulations

All reagents were from Sigma-Aldrich and were ACS grade or better. All cell culture and biochemical manipulations involving heliobacteria, except culture inoculation, were performed anaerobically at ambient temperature using a Coy Type A anaerobic chamber (Coy Laboratory Products, MI, USA) containing an atmosphere of 4% H_2_ / 96% N_2_. Glassware, plasticware, etc. were placed in the chamber 24 h prior to use; solutions were either degassed and placed in the chamber 24 h prior to use or prepared inside the anaerobic chamber using previously degassed water. All solutions were tested for the presence of oxygen using a resazurin-containing indicator (Willis 1991). Manipulations involving *Synechococcus* or chloroplasts were performed aerobically. All spectroscopic experiments were performed at room temperature.

### Cell growth

*Heliomicrobium modesticaldum* was grown in Bellco Hungate anaerobic culture tubes (Bellco no. 2047 − 00125) containing 12 mL of PYE medium (ATCC medium no. 2061); cell culture media was prepared anaerobically as above and Hungate culture tubes containing media and headspace were sterilized via autoclaving after filling. Fresh media was inoculated using the Hungate technique (Hungate 1969; Macy et al. 1972) and cultures were grown in a water bath maintained at 49–50 °C under a bank of eight fluorescent lights. Cell growth was measured at 600 nm. Cultures were grown to late-exponential phase (typically 20–24 h) and harvested via centrifugation at 10,000x*g* for 20 min. at 4 °C. The resulting pellet was resuspended in a minimal volume of 50 mM MOPS pH 7 using a Wheaton Potter-Elvehjem homogenizer (Wheaton no. 358005), supplemented with glycerol to a final concentration of 10% v/v, and stored at −80 °C until use, whereupon it would be thawed for 20 min. and re-homogenized before use; use of the homogenizer ensured a homogenous suspension without damaging the cells. Control experiments indicated that this process did not alter native P800 oxidation kinetics nor (bacterio)chlorophyll compositions but was necessary to obtain consistent results for heliobacterial samples treated with charged redox reagents (Price and Romberger, micropublication in preparation). Cell suspensions of *Synechococcus* sp. PCC 7002 were generously provided by the lab of John Golbeck.

### Chloroplast isolation

Intact chloroplasts were isolated from store-bought spinach according to Joly and Carpentier (2011) with the following modifications. All buffers were prepared as described therein. The composition of Buffer B, used in several procedures and defined here for convenience, is as follows: 50 mM HEPES - NaOH pH 7.6, 0.33 M sorbitol, 2 mM EDTA, 1 mM MgCl_2_, 1 mM MnCl_2_, 10 mM KCl, 1 mM NaCl. Crude extract was obtained from deveined spinach leaves via grinding with mortar and pestle instead of a kitchen blender. The crude extract was then filtered twice through two layers of Miracloth (Sigma-Aldrich no. 475855) and then centrifuged at 200x*g* for 2 min. The resulting supernatant was filtered through a double layer of Miracloth and centrifuged at 750x*g* for 60 s. The resulting supernatant was carefully discarded without disturbing the pellet and the pellet gently resuspended in 20–30 mL of Buffer B. The resuspension was centrifuged at 750x*g* for 60 s., the supernatant carefully discarded, and the pellet gently resuspended in a minimal volume of Buffer B to yield the final suspension of intact chloroplasts. Intactness was determined by a ferricyanide reduction assay (Joly and Carpentier 2011; Lilley et al. 1975) that had been adapted for use on the JTS-10 spectrometer. Lysis of chloroplasts by osmotic shock during the assay was confirmed by an increase in the A_680_/A_550_ ratio (Karlstam and Albertsson 1970). In our hands, the modifications to the chloroplast preparation resulted in a higher percentage of intact chloroplasts at the expense of a decrease in yield and a longer isolation procedure (Gullet, Giudici, and Romberger, unpublished results).

### Spectroscopic measurements

Spectroscopic measurements were conducted using a JTS-10 spectrometer (BioLogic, Claix, France). The instrument was operated in “dark pulse” mode, with detection pulses of 10 µs duration occurring when the actinic light was transiently turned off for 45 µs; detection pulses were emitted every 0.5–100 ms. Detection wavelengths were selected using interference filters (FWHM ≤ 10 nm) placed immediately after detection LEDs and before the focusing lens by virtue of a homebuilt filter holder. P800 redox kinetics were monitored at 800 nm while P700 redox kinetics were monitored at 820 nm. Actinic light was provided by orange-red LEDs centered at approx. 630 nm or far red – near infrared LEDs centered at approx. 720 nm; actinic intensities were measured at the surface of the cuvette using an Apogee Instruments S2-141/44X Series PAR – FAR Red Sensor and are indicated in the text. Reference and sample detectors were protected from actinic and stray light by appropriate long-pass filters. Samples containing chloroplasts were prepared in isotonic Buffer B. Samples of *H. modesticaldum* and *Synechococcus* were prepared using 50 mM MOPS - NaOH pH 7.0; while hypotonic, we observed no evidence of cell lysis and control experiments in which NaCl was used to vary the solution osmolarity showed no changes in P800 kinetics. Samples were diluted to OD < 1.0 based upon the Q_y_ band of the dominant pigment (680 nm for *Synechococcus* and chloroplasts, 788 nm for *H. modesticaldum*) using the appropriate buffer and supplemented as indicated in the text. Samples were prepared in a sealed cuvette (1 cm pathlength) in the dark and were allowed to incubate at room temperature in darkness for 5 min after balancing the detectors and between measurements; samples were prepared aerobically or anaerobically as described above. In experiments where actinic intensities were varied, one sample was used for one set of intensities with the order of intensities randomized to minimize any systematic errors. Percent recovery was calculated by taking the difference between the maximum bleaching and the bleaching immediately before the actinic light was switched off and dividing that difference by the maximum bleaching. For Michaelis-Menten plots, initial rates of P800 or P700 oxidation were estimated by fitting the initial portion of a kinetic trace with a 2nd order polynomial and setting the first derivative of that fit equal to 0. As the initial portions of the kinetic traces were non-linear, this approach allowed a larger portion of the kinetic trace to be fit and avoided inaccurate rates derived from single-point linear fits or from force fitting a linear trendline to curved data (Halling 2020). All data fitting and analysis was performed in Microsoft Excel; data following hyperbolic or sigmoidal trends were fit via least-squares analysis using the Solver add-on (Harris 1998; Dias et al. 2014).

## Results

### Characterization of P800 oxidation kinetics under constant illumination

**Fig. 1:**
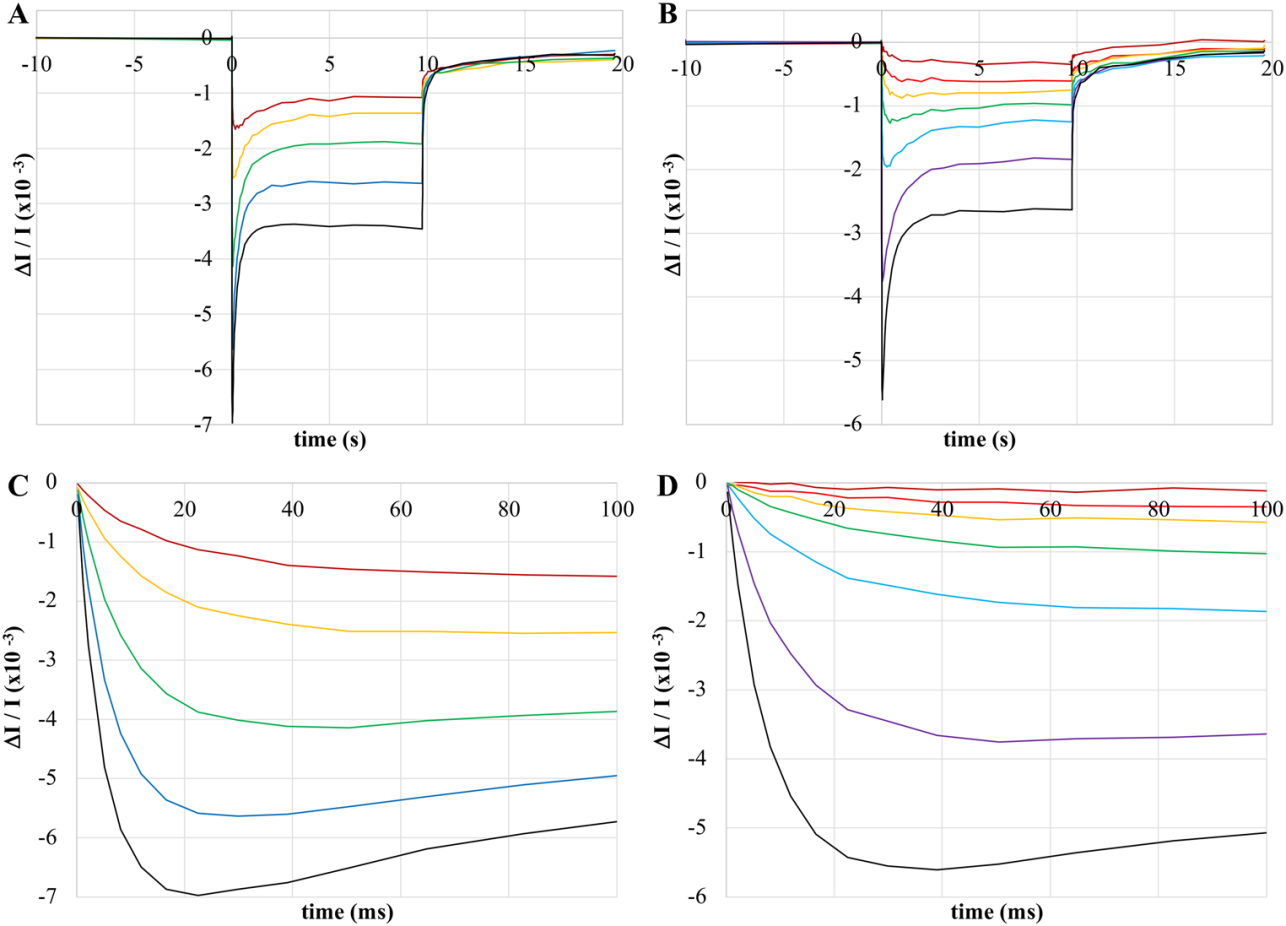
Light-induced absorbance changes, measured at 800 nm, depicting the P800 oxidation of untreated *H. modesticaldum* whole cells at various actinic intensities. Actinic illumination, initiating at 0 s and ending at approx. 9.8 s, was provided by red LEDs centered at 720 nm (**A**,** C**) or orange LEDs centered at 630 nm (**B**,** D**). **C** and **D** are re-plots of **A** and **B**, respectively, depicting the initial kinetics after the onset of actinic illumination. All data was normalized to the same OD_788_. Red actinic intensities were 42 (red), 85 (yellow), 196 (green), 378 (blue), and 607 (black) µE m^− 2^ s^− 1^. Orange actinic intensities were 5 (red), 13 (orange), 25 (yellow), 48 (green), 105 (blue), 314 (purple), and 698 (black) µE m^− 2^ s^− 1^

**Fig. 2:**
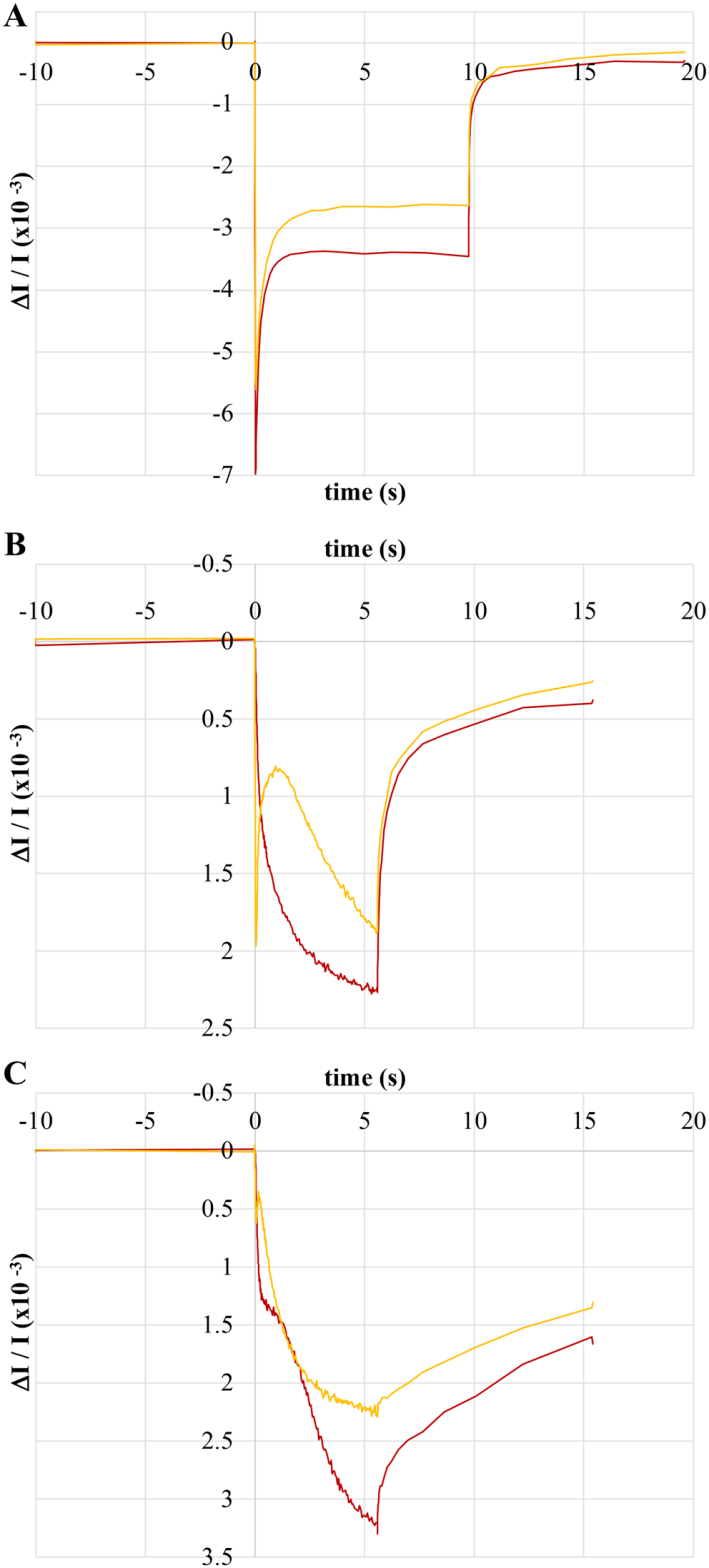
Light-induced absorbance changes depicting P800 oxidation of *H. modesticaldum* whole cells (**A**), P700 oxidation in *Synechococcus* sp. PCC 7002 whole cells (**B**), and P700 oxidation in intact spinach chloroplasts (**C**). Actinic illumination, initiating at 0 s and ending at approx. 9.8 s (**A**) or approx. 5.6 s (**B** and **C**) was provided by red LEDs at 607 µE m^− 2^ s^− 1^ (red traces) or orange LEDs at 698 µE m^− 2^ s^− 1^ (orange traces). Kinetics in each panel were normalized to the same OD_788_. Note the reversed y-axis in **B** and **C** to aid in comparison with **A**

When subjected to continuous illumination, whole cells of *H. modesticaldum* produce complex P800 oxidation kinetics (see Figs. 1 and 2A). Under typical conditions, kinetic traces show three distinct phases during illumination:

- an initial bleaching, reaching a maximum magnitude at 10–30 ms and corresponding to the oxidation of P800 to P800^+^;
- a subsequent light-recovery phase, in which some of the initial bleaching is lost and corresponding to the reduction of a portion of the initially generated P800^+^;
- and a final equilibrium phase, typically occurring 2–3 s after the start of illumination, in which the absorbance change obtains a steady-state and corresponding to a steady-state level of P800^+^.

When the actinic light is turned off, the signal returns towards baseline over the course of a few seconds (although the signal does not always completely recover during the observation timeframe). As shown in Fig. 1, this pattern was observed at all actinic intensities for far red – near IR actinic light (peaking at 720 nm and overlapping with the red absorbance band of whole cells, see Fig. 1A, referred to as red actinic hereafter) and at all but the lowest intensities for orange – red actinic light (peaking at 630 nm, see Fig. 1B, referred to as orange actinic hereafter), although the initial bleaching is somewhat delayed at lower light intensities. As expected, increasing actinic intensities resulted in increasing signal magnitudes, corresponding to increasing P800 oxidation with increasing actinic intensities. These same complex kinetics were observed in whole cells suspended in media or buffer and occurred regardless of whether cells were taken directly from cell culture or used after storage at -80 °C. Control experiments showed that the signals were reproducible with repeated illumination over the course of a few hours (essentially until oxygen penetrated the seal of the cuvette) provided that a sufficient dark incubation period of at least 2 min was allowed between illumination periods. Thus, complex P800 oxidation kinetics, and the light-recovery phase in particular, appear native to heliobacterial photosynthesis and do not appear to be the result of experimental conditions.

While the light-recovery phase was always observed, the magnitude of recovery was variable. On average, samples displayed 54 ± 16% recovery (*n* = 150) at the highest actinic intensities (calculated as the percent of the initial bleaching that was lost during the light recovery phase), although values as low as 11% and as high as 89% were observed. While individual samples from each culture were relatively consistent (percent recovery within a few percent), some variability was observed between cultures. Storage conditions (i.e. cells stored at -80 °C vs. cells obtained directly from culture) were not observed to affect the percent recovery and no correlation between OD_788_ of the sample and percent recovery was found. In combination with the additional results described below, it seems likely that the variability in light-recovery is due to the individual redox state of the culture and the expression level of cyt *c*.

The presence of the light-recovery phase in P800 oxidation kinetics is highly reminiscent of the oscillations in P700 oxidation kinetics reported in cyanobacteria (Yu et al. 1993), algae (Maxwell and Biggins 1976), and pea leaves (Harbinson and Hedley 1993). In these instances, the transient reduction of P700^+^ during constant illumination was largely attributed to rapid donation of electrons to Photosystem I from Photosystem II via the plastoquinone pool and the *b*_*6*_*f* complex (Harbinson and Hedley 1993; Maxwell and Biggins 1977; Yu et al. 1993). Control experiments using whole cells of *Synechococcus* sp. PCC 7002 (Fig. 2B) and freshly isolated spinach chloroplasts (Fig. 2C) produced results similar to those reported previously. When subjected to continuous actinic illumination using orange light (Fig. 2B and C, *orange traces*), which activates both PSI and PSII, P700 oxidation kinetics showed the expected transient light-recovery. When subjected to red actinic light (Fig. 2B and C, *red traces*), which only activates PSI, the transient recovery is lost and replaced by a slower phase of P700 oxidation occurring after the rapid, initial oxidation. Thus, the light-recovery observed in P700 kinetics can be attributed to forward donation of electrons to PSI from PSII. However, such an explanation is not reasonable for P800 oxidation kinetics as the heliobacteria utilize a single Type I reaction center. Therefore, the light-recovery phase in P800 kinetics must have a different origin.

### The effects of redox reagents and inhibitors on P800 oxidation

**Fig. 3:**
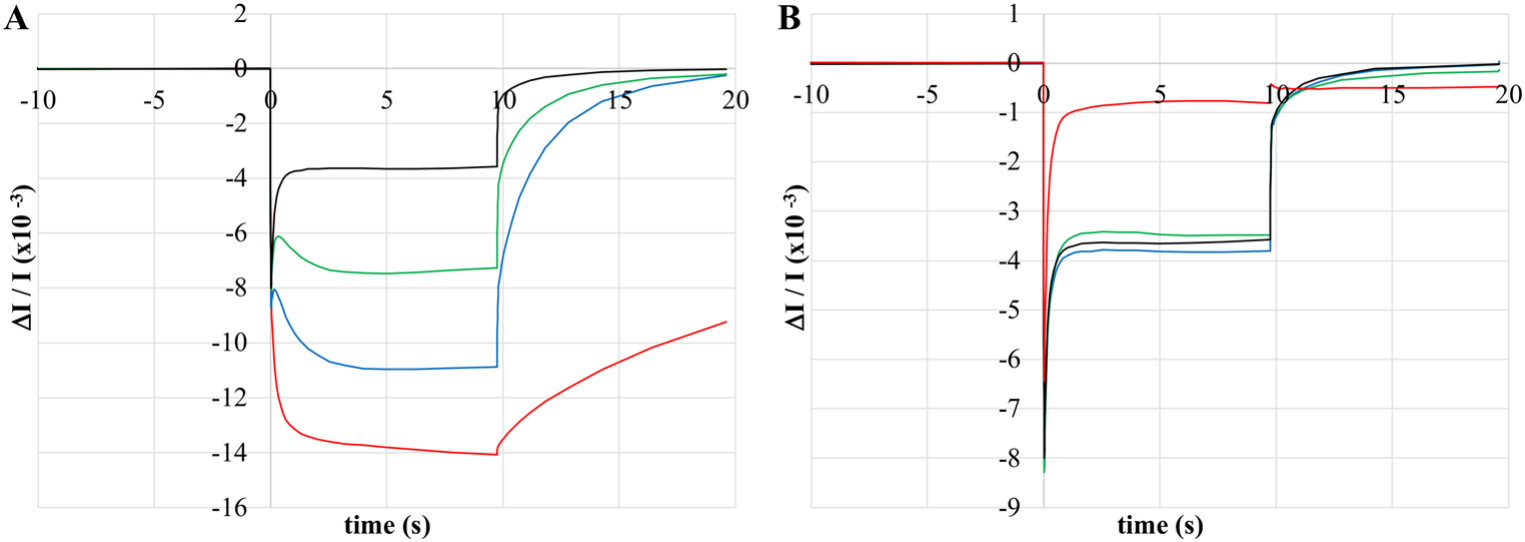
(**A**) Light-induced absorbance changes, measured at 800 nm, depicting P800 oxidation of *H. modesticaldum* whole cells treated with charged oxidants: untreated cells (black trace), ferric nitrate (green trace), ferric ammonium citrate (blue trace), and cupric nitrate (red trace). (**B**) Light-induced absorbance changes, measured at 800 nm, depicting P800 oxidation of *H. modesticaldum* whole cells treated with charged reductant: untreated cells (black trace), sodium thiosulfate (blue trace), sodium ascorbate (green trace), and sodium dithionite (red trace). In both panels, actinic illumination, initiating at 0 s and ending at approx. 9.8 s, was provided by red LEDs at 607 µE m^− 2^ s^− 1^. All oxidants and reductants were at 1.0 mM; all data was normalized to the same OD_788_

The addition of charged redox reagents had pronounced effects on P800 oxidation kinetics. Treatment of whole cells with the oxidants ferric nitrate, copper nitrate, or ferric ammonium citrate resulted in a dramatic increase in signal magnitude and significantly diminished the light-recovery phase (Fig. 3A). Reductants with less reducing potential, such as thiosulfate (*E°’* = +198 mV, Kurth et al. 2015) or ascorbate (*E°’* = -81 mV, Fruton 1934), had little effect on P800 oxidation kinetics while reductants with more reducing potential, such as dithionite (*E°’* = -467 mV at the 1.0 mM concentrations used here, Mayhew 1978), produced effects opposite that of charged oxidants: the initial bleaching was much smaller relative to untreated cells and the light-recovery phase was enhanced, with the steady-state absorbance value nearly returning to baseline (Fig. 3B). In heliobacteria, the immediate donor to the HbRC is a membrane-associated cyt *c* located on the periplasmic face of the membrane (Fuller et al. 1985; Kashey et al. 2014; Redding et al. 2014). As charged compounds cannot cross the cell membrane, and given that we avoided the use of lipophilic redox mediators, these effects are most easily explained as the result of reactions between the added redox reagents and cyt *c*. Charged oxidants could oxidize cyt *c* directly, thereby inhibiting forward donation to the HbRC and preventing the reduction of P800^+^. Strong reductants could act as an additional electron source for cyt *c*, thereby increasing the re-reduction rate of HbRC oxidized cyt *c* and enhancing the rate of P800^+^ reduction.

**Fig. 4:**
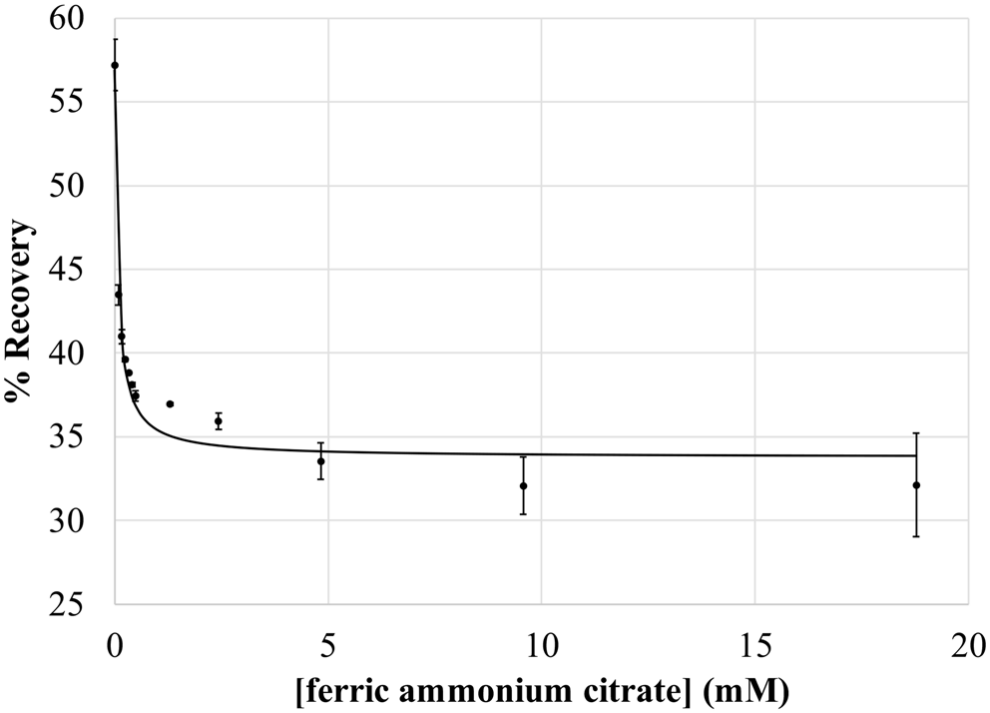
Inhibition of forward donation to P800^+^ by ferric ammonium citrate. Each point is the average of at least three samples treated with ferric ammonium citrate at the indicated concentration with all samples obtained from the same culture; error bars are standard deviations. Kinetics were measured as in Fig. 3; all data was normalized to the same OD_788_. Percent recovery is determined as the difference between the magnitudes of the initial bleaching and steady-state bleaching and expressed as the percentage of initial bleaching. Data was fit (*solid line*) with the equation $\:Y={y}_{min}+\left(\frac{{y}_{max}\:-\:{y}_{min}}{1+\:\frac{C}{{IC}_{50}}}\right)$; where Y is the percent recovery, y_min_ and y_max_ are the minimum and maximum observed percent recovery, C is the FAC concentration, and IC_50_ is the FAC concentration producing 50% inhibition

Given its historic use as a Hill reagent and its limited effect on whole cell integrity and solubility, we further characterized the effect of ferric ammonium citrate (FAC) on P800 oxidation kinetics. As expected, increasing concentrations of FAC resulted in increasing initial bleaching and decreasing values of percent recovery until saturation was achieved, typically around 10 mM FAC. Plots of the percent recovery as a function of FAC concentration (Fig. 4) followed the expected hyperbolic curve for an inhibitor and produced IC_50_ values of 63 ± 11 µM (*n* = 9, IC_50_ is the effector concentration producing 50% of the maximal effect). These values are in line with the IC_50_ values reported for the cytochrome *bc* complex inhibitors azoxystrobin and terbutryn (Kashey et al. 2018), although it is likely that the underlying mechanisms are different; nevertheless, the similar IC_50_ values highlight the effectiveness of FAC as an inhibitor. As with untreated cells, the effect on the light-recovery phase was somewhat variable. At saturating FAC concentrations, treatment with FAC resulted in complete loss of the light-recovery phase in 53% of samples (*n* = 32). For the 47% of samples in which a portion of the light-recovery phase remained, the percent recovery decreased by 74 ± 15% of the initial percent recovery prior to FAC treatment. No correlation was observed between percent recovery prior to FAC treatment and percent recovery after FAC treatment.

The characteristics of the FAC effect can be rationalized by assuming that FAC oxidizes the various cytochromes of the photosynthetic electron transfer chain, thereby inhibiting forward donation to P800^+^. Recent work by Adam, et al. reports a mid-point potential of -30 mV to + 10 mV at pH 7.4 for iron-citrate complexes (Adam et al. 2015) whereas mid-point potentials of + 217 mV for *H. modesticaldum* cyt *c* (Kashey et al. 2014) and + 71 mV for the diheme cytochrome *c*_4_ subunit of the cytochrome *bc* complex (Yue et al. 2012) have been reported. Given these mid-point potentials, FAC could be functioning as an alternative acceptor for the cytochrome *bc* complex, thereby preventing the re-reduction of oxidized cyt *c* and subsequent forward donation to P800^+^. Variations in the redox state of the culture and/or differences in the expression levels of cyt *c* would affect the competition between FAC and cyt *c* for the cytochrome *bc* complex, therefore affecting the amount of reduced cyt *c* available to reduce P800^+^ and, therefore, the magnitude of the light-recovery phase. However, IC_50_ values would not be strongly affected as they are more indicative of the initial binding interaction between FAC and the *bc* complex and less so of electron transfer itself. Alternatively, given the high concentration of FAC relative to cyt *c*, FAC may be oxidizing a portion of the cyt *c* pool directly. Indeed, preliminary studies have suggested that FAC may be affecting both cyt *c* and the cytochrome *bc* complex (Price and Romberger, unpublished results). Additional spectroscopic and enzymological experiments will be needed to differentiate between these possibilities.

**Fig. 5:**
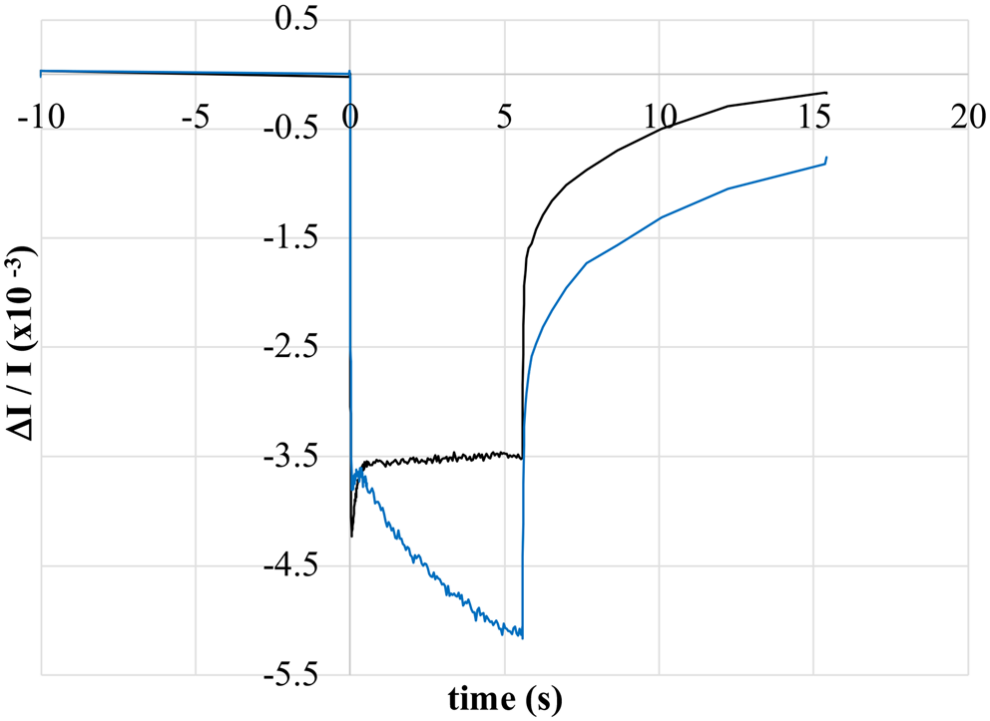
Light-induced absorbance changes, measured at 800 nm, depicting P800 oxidation of *H. modesticaldum* whole cells treated with azoxystrobin (blue trace) or untreated (black trace). All data was normalized to the same OD_788_. Actinic illumination, initiating at 0 s and ending at approx. 5.2 s, was provided by red LEDs at 607 µE m^− 2^ s^− 1^

The hypothesis that the light-recovery phase is a result of fast forward donation to P800^+^ from reduced cyt *c* was tested by treating heliobacterial whole cells with azoxystrobin and measuring the P800 oxidation kinetics. Azoxystrobin has recently been identified as an inhibitor of the heliobacterial *bc* complex (Kashey et al. 2018). Thus, incubation with azoxystrobin should prevent the re-reduction of HbRC oxidized cyt *c*_*553*_, resulting in the loss of the light-recovery phase. As hypothesized, when cells were incubated with azoxystrobin, the light-recovery phase was abolished while the maximum absorbance change increased (Fig. 5). Combined with the effects of charged redox reagents, these results strongly suggest that the light-recovery phase is the result of fast forward donation from reduced cyt *c* to P800^+^ and not the result of charge recombination between P800^+^ and F_X_¯ due to acceptor-side limitations.

### Steady-state kinetics analysis of P800 oxidation

**Fig. 6:**
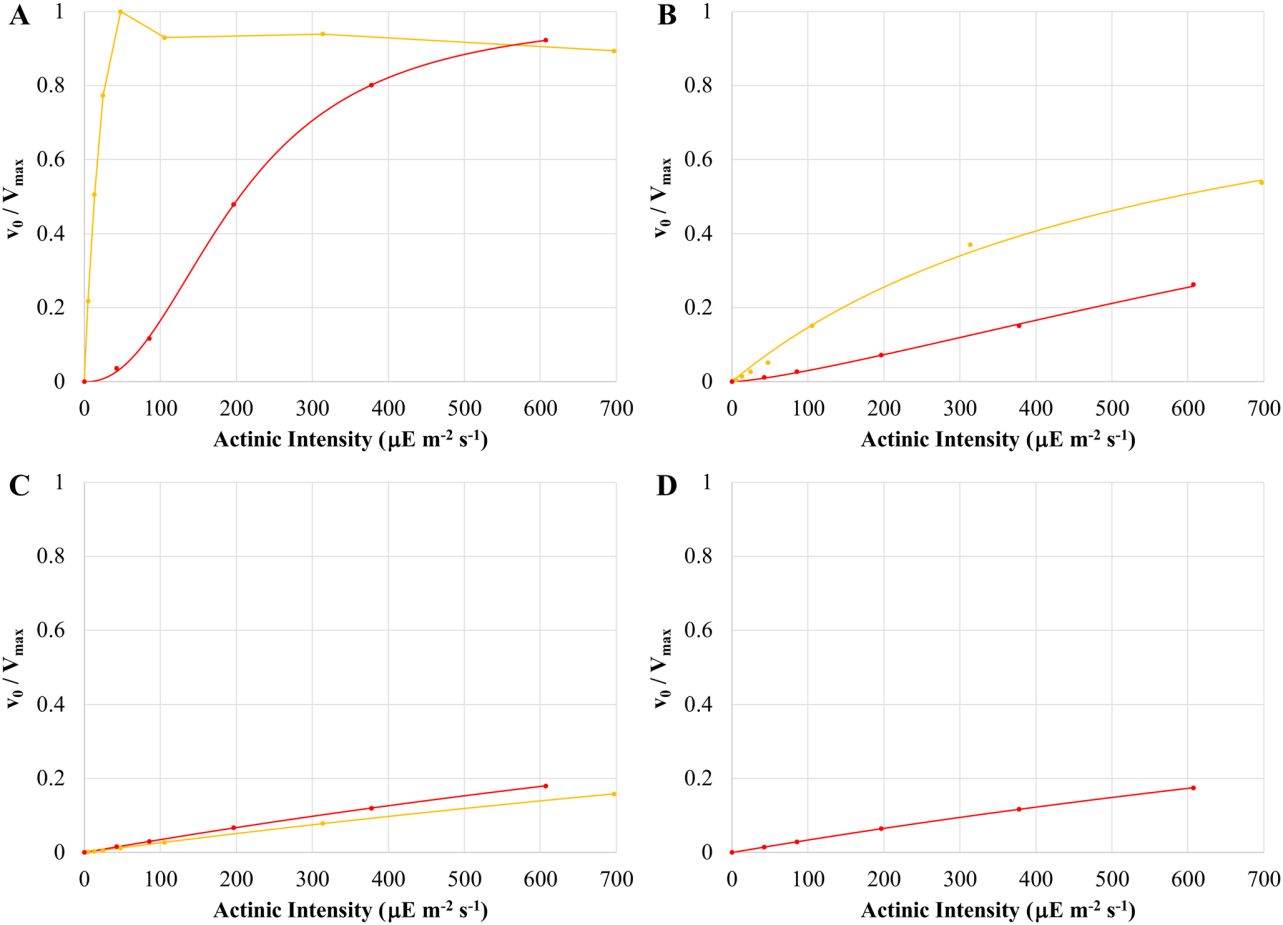
Steady-state kinetics analysis depicting the relationship between the initial rate of primary donor oxidation and actinic intensity: P700 oxidation in intact spinach chloroplasts (**A**), P700 oxidation in whole cells of *Synechococcus* sp. PCC 7002 (**B**), P800 oxidation in untreated whole cells of *H. modesticaldum* (**C**), and P800 oxidation in whole cells of *H. modesticaldum* treated with 19.2 mM ferric ammonium citrate (**D**). Actinic illumination was provided by red LEDs (red trends) or orange LEDs (orange trends) at the intensities indicated. Initial rates (*filled circles*) are normalized to the predicted V_max_ except for chloroplasts under orange illumination, where V_max_ was taken as maximum observed rate due to inhibition at higher intensities. Plots shown are representative data with each set of rates obtained from a single sample. Initial rates are fit with the Michaelis-Menten equation (*solid lines*), except for chloroplasts under orange illumination, which were left unfitted. Kinetic parameters reported in the text are the average of Michaelis-Menten fits on multiple samples. See text for additional details

The continuous illumination conditions employed in this work are more closely akin to classic steady-state enzyme kinetics than the single-turnover flash photolysis experiments typically employed when measuring primary donor oxidation. From this perspective, the HbRC and PSI function as enzymes with three substrates and two products in which light, reduced cytochrome, and oxidized ferredoxin are substrates and oxidized cytochrome and reduced ferredoxin are products. Thus, we analyzed the initial rate of primary donor oxidation as a function of actinic light intensity. As shown in Fig. 6, the resulting trends between whole cells of heliobacteria versus those of intact chloroplasts and whole cells of *Synechococcus* sp. PCC 7002 were strikingly different.

When intact chloroplasts were exposed to orange actinic light, the initial rate of P700 oxidation saturated at light intensities as low as 48 µE m^− 2^ s^− 1^ (Fig. 6A, *orange trace).* Increasing light intensities resulted in somewhat decreasing rates, reminiscent of classic substrate inhibition. Initial rates prior to saturation showed a hyperbolic dependence on actinic intensity, consistent with classic Michaelis-Menten kinetics and producing a K_M_ of 25 ± 5 µE m^− 2^ s^− 1^ (*n* = 5, we retain the use of K_M_ to indicate the actinic intensity resulting in a half-maximal rate in order to distinguish between inhibitor effects, steady-state kinetics, and light-saturation profiles of P800^+^ signal size). When red actinic light was used (Fig. 6A, *red trace*), the initial rate of P700 oxidation showed a sigmoidal dependence on actinic intensity. Fitting the data with the Michaels-Menten equation adjusted for sigmoidal kinetics produced a K_M_ of 230 ± 20 µE m^− 2^ s^− 1^ and a Hill coefficient of 2.2 ± 0.7 (*n* = 5). The higher K_M_ relative to orange light is not unexpected given the weak absorbance of far-red light by chloroplasts. The initial rate of P700 oxidation in whole cells of *Synechococcus* sp. PCC 7002 showed a similar dependence on actinic intensity as intact chloroplasts (Fig. 6B), although K_M_ values were higher for both actinic sources (900 ± 300 µE m^− 2^ s^− 1^, *n* = 6 for orange light and 1000 ± 500 µE m^− 2^ s^− 1^, *n* = 6 for red light) and the Hill coefficient for red actinic light was smaller (1.41 ± 0.19, *n* = 6). As a result, saturation of the initial rate was not obtained for these samples. Nevertheless, assuming an intensity of 1800 µE m^− 2^ s^− 1^, the initial rate of P700 oxidation for both intact chloroplasts and cyanobacterial whole cells should be saturated or mostly saturated with typical levels of sunlight.

In contrast to P700 oxidation kinetics, the initial rate of P800 oxidation (Fig. 6C) showed only a weak hyperbolic dependence on actinic intensity and no saturation was observed, suggesting that our experiments were conducted well below the K_M_. Indeed, observed rates at the highest available red actinic intensities averaged 18 ± 6% of the estimated V_max_ values and never exceeded 35% (*n* = 35), while the highest observed rates with orange actinic light averaged only 13 ± 7% of the estimated V_max_ values (*n* = 3) with several additional plots producing unreasonable fits. The K_M_ values were correspondingly large, with a value of 2700 ± 900 µE m^− 2^ s^− 1^ for red actinic light (*n* = 35) and 6000 ± 5000 µE m^− 2^ s^− 1^ for orange actinic light (*n* = 3). These values must be taken with caution given the limited portion of the Michaelis-Menten curve we were able to measure. As red actinic light better overlaps the red absorbance band of the *H. modesticaldum* absorbance spectrum, the preference for red light is not unexpected. Notably, these K_M_ values are an order of magnitude higher than those observed with chloroplasts and *Synechococcus* and no rate saturation was observed, indicating that the HbRC is operating in a much more light-limited capacity than its Photosystem I counterpart.

Treating heliobacterial whole cells with FAC to inhibit forward donation to P800^+^ produced similar kinetics as untreated cells (Fig. 6D). Using red actinic light, measured rates at the highest available intensities averaged 18 ± 3% and never exceeded 23% of the estimated V_max_ values while the K_M_ remained large at 2800 ± 500 µE m^− 2^ s^− 1^ (*n* = 18), a value that is not statistically different than that obtained in untreated cells. Therefore, the very high light requirement does not appear to be a result of fast forward donation from cyt *c* limiting the net rate of P800 oxidation. A simple argument pointing to the limited overlap of the actinic light with the absorbance spectrum of *H. modesticaldum* is also problematic. P700 oxidation rates using red actinic light, which has minimal overlap with the Photosystem I absorbance spectrum, could be saturated or near saturated with typical sunlight intensities. However, it would take the light of several suns to saturate the initial rate of P800 oxidation under these same conditions. Thus, it appears that heliobacterial phototrophy is significantly light-limited compared to other systems and that the observed light-limitation is inherent to the HbRC.

### Light-saturation profiles estimating the percentage of P800 oxidized under constant illumination

The oxidation kinetics after the light-recovery phases are starkly different between P800 and P700. In P800 oxidation kinetics (Fig. 2A), the light-recovery phase results in a steady-state pool of P800^+^ that is much smaller than that initially obtained. This is opposite that of P700 oxidation kinetics (Fig. 2B and C), where the light-recovery phase is transient and results in a larger steady-state pool of P700^+^ than that initially obtained.

The above observation, the complimentary finding that treatment with FAC and other compounds resulted in increased bleaching, and steady-state kinetics consistent with severe light-limitation, suggested that a significant portion, if not majority, of the P800 pool remained in the reduced state during constant illumination. As such, we investigated the combined effects of actinic intensity and FAC on the signal magnitudes observed during P800 oxidation. Control experiments indicated that the P800 oxidation signal magnitudes were proportional to the optical density of samples at 788 nm, thus signal magnitudes between samples were normalized to the largest OD_788_. In order to minimize the variations noted in our preceding experiments, samples were only compared between cultures grown at the same time.

**Fig. 7:**
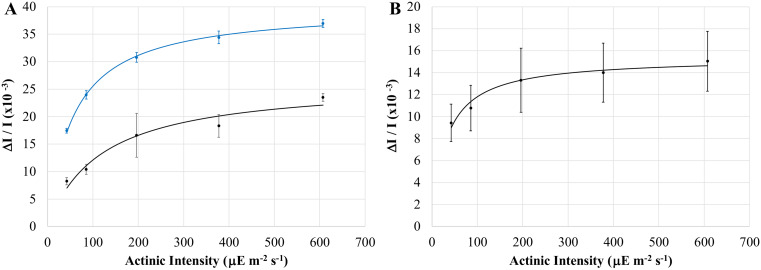
Light saturation profiles depicting the effect of actinic intensity on P800^+^ signal magnitude for the initial P800 bleaching (**A**) and steady-state P800 bleaching (**B**); untreated cells in black, cells treated with 19.2 mM ferric ammonium citrate in blue. The y-axis is shown as the absolute value of the signal magnitude for clarity. Each point is the average of at least three samples with error bars depicting standard deviation; all data was obtained from a single culture and normalized to the same OD_788_. Data is fit (*solid lines*) using the equation B = (I•B_max_) / (I + K_50_), where B is the observed signal magnitude, I is the actinic intensity, B_max_ is the maximum signal magnitude under saturating actinic intensities, and K_50_ is the actinic intensity producing 0.5 B_max_

As seen in Fig. 7A, the maximum initial bleaching, which represents the maximum amount of P800 that is oxidized, has a strong hyperbolic dependence on actinic intensity for both untreated and FAC-treated cells. Fitting these light-saturation profiles with the classic Hill equation allowed for the determination of B_max_, which represents the maximum possible bleaching under saturating actinic light. As with our previous observations, treatment with FAC resulted in increased signal magnitudes corresponding to a dramatic increase in B_max_ relative to untreated cells, with B_max_ values in untreated cells accounting for only 75% ± 8% (*n* = 17) of the B_max_ in FAC-treated cells. Thus, FAC-treatment allows for oxidation of a much larger portion of the P800 pool.

Steady-state bleaching, occurring after the light-recovery phase has reaching equilibrium and measured immediately before the actinic light was switched off, represents the amount of P800 oxidized under constant illumination. Steady-state bleaching demonstrated a weak dependence on actinic intensity (Fig. 7B), with resulting B_max_ values taken to represent the amount of P800 oxidized under constant illumination. If the B_max_ of FAC-treated cells represents the entirety of the P800 pool, then the ratio of the two values would indicate the percentage of P800 that is oxidized under continuous illumination. Consistent with the light-limited model suggested above, it appears that a mere 32% ± 7% (*n* = 17) of the P800 pool is oxidized during the continuous illumination conditions that occur during normal cell growth.

## Discussion

In this work, we have presented an initial characterization of the complex P800 oxidation kinetics occurring in whole cells of *H. modesticaldum* when exposed to continuous illumination. Immediately upon illumination, a rapid bleaching at 800 nm occurs, corresponding to the oxidation of P800 to P800^+^. The bleaching reaches a maximum between 10 and 30 ms, after which a slower recovery phase (termed the light-recovery phase) occurs, during which time the rate of P800^+^ reduction outpaces that of P800 oxidation. While the magnitude of the light-recovery phase seems variable, it typically accounts for 50–60% of the initial pool of oxidized P800. An equilibrium between P800 oxidation and P800^+^ reduction is typically reached by 2–3 s, at which point the total absorbance change is stable until the actinic light is turned off and the signal returns to baseline. The presence of a light-recovery phase was surprising as it is reminiscent of P700 oxidation kinetics when cyanobacteria (Yu et al. 1993), algae (Maxwell and Biggins 1976), and pea leaves (Harbinson and Hedley 1993) are exposed to continuous white light. In these organisms, the transient light-recovery phase was the result of forward donation of electrons from Photosystem II to Photosystem I. Using intact chloroplasts isolated from spinach and whole cells of *Synechococcus* sp. PCC 7002, we were able to reproduce the reported P700 oxidation kinetics and, by using red actinic light that only activates Photosystem I, confirm that the transient light-recovery phase is attributable to forward donation originating from Photosystem II. To our knowledge, this is the first report of P700 oxidation kinetics under these conditions in spinach chloroplasts. Observation of a light-recovery phase in both organisms suggests that it is a common feature of phototrophy driven by a pair of Type I and Type II RCs working in tandem. However, the heliobacteria utilize a single Type I RC to drive phototrophy, thus the light-recovery phase in *H. modesticaldum* must have a different biochemical origin. Treatment of whole cells of *H. modesticaldum* with charged redox reagents – in particular FAC – and the cytochrome *bc* complex inhibitor azoxystrobin confirmed that the light-recovery phase in P800 oxidation kinetics was due to fast forward donation to P800^+^ from cyt *c* and likely not from charge recombination between P800^+^ and F_X_¯.

A steady-state kinetics analysis measuring the effect of actinic intensity on the initial rate of primary donor oxidation revealed stark differences between P700 and P800 oxidation and suggested that heliobacterial phototrophy is light limited. Regardless of the actinic source or the sample organism, initial rates of P700 oxidation followed classic models of enzyme kinetics (either Michaelis-Menten or Hill kinetics) with rates saturating at actinic intensities equivalent to or well-below those of full sunlight. While initial rates of P800 oxidation retained the hyperbolic dependence on actinic intensities, the estimated K_M_ values were a full order of magnitude higher than those of P700 oxidation, suggesting that rate saturation would require actinic intensities equivalent to several suns. Removal of fast forward donation by treatment with FAC did not significantly change the K_M_ values of P800 oxidation, suggesting that light-limitation is inherent to the HbRC.

In support of a light-limited model, we determined that a majority of the P800 pool remains reduced during continuous illumination, with a mere 32% of the P800 pool becoming oxidized. This is starkly different than the observed P700 oxidation kinetics, where continued illumination results in oxidation of a much larger portion of the P700 pool. Thus, it seems that a fundamental difference between heliobacterial phototrophy and oxygenic photosynthesis is the redox state of the Type I RC primary donor pool under continuous illumination: in heliobacteria, a majority of the primary donor pool remains reduced whereas, in oxygenic phototrophs, a majority of the primary donor pool becomes oxidized.

One alternative to our light-limited model would be acceptor-side limitation. Studies by Collins et al. (2010) and Redding et al. (2014) have argued that fluorescence from whole cells of heliobacteria is due to acceptor-side limitation. Under conditions of high-light, the acceptor pool becomes reduced while forward donation from cyt *c* continues, leading to HbRCs in a P800^+^ A_0_^−^ F_X_^−^ state. Subsequent recombination between P800^+^ and A_0_^−^ results in reduced P800 in the singlet excited state, which relaxes to the ground state by the observed fluorescence. Indeed, these studies confirmed an acceptor-side limited mechanism by utilizing dithionite in combination with lipophilic redox mediators (resazurin in Collins, et al. and phenazine methosulfate in Redding, et al.), which allowed reducing equivalents to cross the cell membrane and reduce the intracellular acceptor pool. In our absorbance-based experiments, rapid recombination between P800^+^ and A_0_^−^ due to acceptor-side limitation could appear as the observed light-recovery phase and a very high K_M_ in our steady-state kinetics. Although our study avoided the use of lipophilic redox regeants in an effort to localize redox effects to the extracellular side the membrane, if dithionite was able to cross the membrane due to either a higher than expected permeability or via a currently unidentified transporter, then treatment of cells with dithionite would reduce the acceptor pool and result in the observed increase in the light-recovery phase. While acceptor-side limitation would explain our observations regarding a light-recovery phase and treatment with dithionite, it does not account for the effects of azoxystrobin which, as a *bc* complex inhibitor, argues that the light-recovery phase arises due to rapid forward donation from the cytochrome pool. Moreover, a plethora of acceptors to the HbRC have recently been discovered, with at least three different ferredoxins having been shown to accept electrons from F_X_ (Walters et al. 2024). In addition, the finding that cyanobacterial flavodoxin also accepts electrons from F_X_ (Romberger and Golbeck 2012) suggests that still other acceptor proteins may function in vivo. Finally, there is significant evidence supporting a secondary electron transfer pathway involving membrane-soluble menaquinone (Kashey et al. 2018). Indeed, this incongruity between a seemingly large acceptor pool and acceptor-limitation was also noted in the 2014 study by Redding, et al. However, given questions regarding the permeability of dithionite for the cell membrane, the present work cannot completely exclude acceptor-side limitation.

While our results most strongly argue for a light-limited model of heliobacterial phototrophy, our steady-state kinetics approach was unable to determine the mechanism of that light-limitation. One possibility would be poor transfer of excitation energy from the antennae to P800, which would result in a requirement for high intensity actinic light. However ultrafast spectroscopic (Kojima et al. 2020) and theoretical studies (Kimura et al. 2021) have indicated that energy equilibration in the antennae and energy transfer from the antennae to P800 is efficient. A second possibility is that much of the actinic light is not absorbed by the antennae of the HbRC. There is some support for this second possibility in our experiments as intact chloroplasts and whole cells of *Synechococcus*, which possess large antennae systems, demonstrated saturation of P700 oxidation rates whereas heliobacteria, which possess much smaller antennae systems, demonstrated light-limited P800 oxidation rates. Further work will be needed to differentiate between these, or other possible mechanisms.

Except for some differences in wild-type kinetics, our results are rather consistent with the continuous illumination kinetics reported by Leung et al. (2021) on their studies of *petCBDA* knockouts in *H. modesticaldum*. In both sets of studies, inhibition of forward donation either through the use of charged oxidants, *bc* complex inhibitors, or deletion of the *bc* complex entirely, resulted in a much larger amount of oxidized P800. These observations argue for a light-limited system in which the generation of P800^+^ is relatively slow, such that a significant portion of the P800 pool remains unoxidized. One notable difference between the two studies is the absence of the light-recovery phase in the study by Leung, et al. While our lab has always observed a light-recovery phase, the percent recovery is variable between cultures. Thus, one possible explanation for the discrepancy could be cell culture conditions. Although media formulations and growth temperatures between the studies were similar, the light sources are different (warm fluorescent light vs. near infra-red), as is the absence or presence of CO_2_. Experimental conditions would not have a significant effect as instrumentation is nearly identical and the presence of a light-recovery phase is observed in both cells suspended in culture media and those resuspended in buffer.

The light-limited heliobacterial system stands in stark contrast to other known photosynthetic systems, many of which utilize extensive antennae to increase the number of absorbed photons. Like the heliobacteria, the environmental niches of the chloracidobacteria and green sulfur bacteria also limit the number of available photons. Yet these organisms, which also exclusively use a homodimeric Type I RC, utilize chlorosomes to improve their light-harvesting capabilities (Bryant and Frigaard 2006). Given these examples, the light-limited system of the heliobacteria seems counter-intuitive. However, there are potential advantages. By limiting the light-driven charge-separation reaction while maintaining fast donor-side and acceptor-side reactions, a light-limited system would maximize the likelihood of reducing equivalents entering metabolism and minimize the likelihood of reducing non-physiologically useful acceptors. Hence, many of the photoprotective systems evolved by other phototrophs to scavenge reactive oxygen species and other highly reactive photochemical side-products would be unnecessary. Moreover, a light-limited system would utilize fewer photosynthetic pigments and the proteins associated with binding those pigments, thereby lowering the metabolic, genetic, and regulatory costs of utilizing and maintaining the reaction center and associated antennae proteins. Thus, the heliobacteria may be in the unique position where the metabolic advantages of faster light-driven reactions are outweighed by the metabolic costs of building, protecting, and regulating such a system.

A number of characteristics now point to the unusual nature of heliobacterial phototrophy including pigment composition, use of the simplest Type I RC, the redox state of the primary donor pool under continuous illumination, and the very high-light intensities necessary to saturate the rate of primary donor oxidation. While potentially limited in its ability to elucidate mechanism, the steady-state kinetics approach utilized here was essential to characterizing the light-limitation of heliobacterial phototrophy. Continued characterization will not only shed light on photosynthetic electron transfer in the heliobacteria but could also identify previously unrecognized effectors in other phototrophs or give insight into their phototrophic mechanisms. Given their unique characteristics, the heliobacteria remain an important counterpoint in the broader understanding of photosynthesis.

## Data Availability

No datasets were generated or analysed during the current study.
